# Identification of Co-expressed Genes Between Atrial Fibrillation and Stroke

**DOI:** 10.3389/fneur.2020.00184

**Published:** 2020-03-24

**Authors:** Yan-fei Zhang, Ling-bing Meng, Meng-lei Hao, Jie-fu Yang, Tong Zou

**Affiliations:** ^1^Department of Cardiology, Beijing Hospital, National Center of Gerontology, Beijing, China; ^2^Neurology Department, Beijing Hospital, National Center of Gerontology, Beijing, China; ^3^Department of Geriatric Medicine, Affiliated Hospital of Qinghai University, Xining, China

**Keywords:** atrial fibrillation, stroke, bioinformatic technology, differentially expressed genes, hub genes

## Abstract

Atrial fibrillation (AF) increases the risk of ischemic stroke and systemic arterial embolism. However, the risk factors or predictors of stroke in AF patients have not been clarified. Therefore, it is necessary to find effective diagnostic and therapeutic targets. Two datasets were downloaded from the Gene Expression Omnibus (GEO) database. Differently expressed genes (DEGs) were identified between samples of atrial fibrillation without stroke and atrial fibrillation with stroke. Enrichment analysis of Gene Ontology (GO) and Kyoto Encyclopedia of Genes and Genomes (KEGG) by Gene Set Enrichment Analysis (GSEA), construction and analysis of protein-protein interaction (PPI) network and significant module, and the receiver operator characteristic (ROC) curve analysis were performed. A total of 524 DEGs were common to both datasets. Analysis of KEGG pathways indicated that the top canonical pathways associated with DEGs were ubiquitin-mediated proteolysis, endocytosis, spliceosome, and so on. Ten hub genes (SMURF2, CDC42, UBE3A, RBBP6, CDC5L, NEDD4L, UBE2D2, UBE2B, UBE2I, and MAPK1) were identified from the PPI network and were significantly associated with a diagnosis of atrial fibrillation and stroke (AFST). In summary, a total of 524 DEGs and 10 hub genes were identified between samples of atrial fibrillation without stroke and atrial fibrillation with stroke. These genes may serve as the target of early diagnosis or treatment of AF complicated by stroke.

## Introduction

Atrial fibrillation (AF) is a type of supraventricular tachyarrhythmia characterized by rapid and disordered atrial electrical activity (1). The atrium loses effective contraction due to disordered electrical activity and the atrioventricular node presents diminished conduction to rapid atrial activation, resulting in an extremely irregular ventricular rhythm and a rapid or slow ventricular rate, which leads to decreased cardiac blood pumping function and mural thrombosis formation in the atria (2, 3). Stroke is an acute cerebrovascular disease, which is a group of diseases that causes brain tissue damage due to the sudden rupture of blood vessels in the brain or vascular occlusion preventing blood from flowing into the brain (4, 5). AF increases the risk of stroke, with incidence rates of 1.92% a year. Compared with non-AF-related strokes, strokes caused by AF have a worse prognosis, with a mortality rate of nearly 20% and a disability rate of nearly 60% (6). However, the molecular mechanisms of strokes caused by AF are unclear (7).

With the development of molecular biology and second-generation sequencing technology, it is possible to explore the pathogenesis of diseases on a large scale at the gene and molecular level (8). Bioinformatics analysis can obtain a large amount of gene expression information simultaneously and explore differentially expressed genes (DEGs) related to disease initiation and progression (9). Concurrently, these DEGs also provide a novel direction for the diagnosis and treatment of diseases (10). By studying the difference in gene expression profiles between AF without strokes and AF-related strokes, Allende et al. (11) suggested that Hsp70 protects AF-related stroke patients via the prevention of thrombosis without augmenting the risk of bleeding, and it might be a novel biomarker to cure patients of stroke caused by AF. Stamova et al. (12) tried to explore the distinction of gene expression in the cardioembolic stroke patients and advocated that future research should be designed to verify the role of DEGs in strokes and AF. Studying the genetic factors of stroke caused by AF is of great significance, and the research is vital to understand the pathogenesis and provide a theoretical basis of molecular genetics for the precise treatment.

Therefore, in this research, two datasets, GSE66724, and GSE58294, were downloaded from the Gene Expression Omnibus (GEO), followed by screening and enrichment of DEGs and identification of hub genes. Finally, the study reviews diagnostic and prognostic information provided by hub genes and discusses the potential value of hub genes as a new therapeutic target for patients with AF-stroke.

## Materials and Methods

### Access to the Data

Gene Expression Omnibus (GEO) (13) is a gene expression data warehouse and online resource for retrieving gene expression data from any species or artificial source. GEO mainly contains a variety of chip data and some sequencing data. Two datasets [GSE66724 (11) and GSE58294 (12)], which were all annotated in the platform of GPL570 [HG-U133_Plus_2] Affymetrix Human Genome U133 Plus 2.0 Array were downloaded from the GEO database. GSE66724 consisted of 16 whole blood samples, which were taken from eight patients with atrial fibrillation and without stroke (AF, *n* = 8), and eight patients with atrial fibrillation and stroke (AFST, *n* = 8). The subject characteristics of individuals in the GSE66724 were showed in the Table 1 (11). GSE58294 also included a total of 92 whole blood samples, which were taken from 23 patients with atrial fibrillation and without stroke (AF, *n* = 23) and 69 patients with atrial fibrillation and stroke (AFST, *n* = 69). However, the lack of subject characteristics of individuals in the GSE58294 was a limitation and the patients with stroke were in the hyperacute phase.

**Table 1:** Clinical characteristics of the individuals in the GSE66724.

	**AF without stroke** **(*n* = 8)**	**AFST (*n* = 8)**	***P***
Age (years)	68.5 (61.3–77.8)	70.0 (65.3–77.5)	0.598
Sex (female), n (%)	3 (37.5)	3 (37.5)	0.999
CHAD	1 (1–2)	1 (1–2)	0.626
Hypertension, n (%)	6 (75.0)	5 (62.5)	0.619
Congestive heart failure, n (%)	2 (25.0)	1 (12.5)	0.554
Diabetes, n (%)	2 (25.0)	3 (37.5)	0.619
Leukocytes (x10^3^, cells/ml)	6.70 (6.35–8.88)	7.60 (6.50–8.23)	0.958
Neutrophils (%)	4.10 (3.35–6.35)	4.13 (3.41–5.25)	0.958
Monocytes (%)	1.98 (1.69–2.29)	2.40 (1.92–2.53)	0.189
Lymphocytes (%)	0.71 (0.41–0.91)	0.59 (0.51–0.64)	0.958
Statins, n (%)	3 (37.5)	3 (37.5)	0.999
Anti-hypertensive drugs, n (%)	4 (50.0)	4 (50.0)	0.999

*Values for age, CHAD, leukocytes, neutrophils, monocytes, and lymphocytes are the median [interquartile range (IQR)]. CHAD is the CHADS_2_ index after subtracting the previous stroke punctuation. Neutrophils, monocytes, and lymphocytes are expressed as percentage of the total number of leukocytes*.

### The Repeatability Test for the Intra-Group Data

The repeatability of data was verified by Pearson's correlation test. R (14) is an open language and environment for statistical computing and mapping, which was maintained by a large and active global research community. Pearson's correlation test and the mapping of heatmaps were completed by the R language. Principal component analysis (PCA) (15), a strong mathematical method, was capable of reducing the data's complexity. The PCA could capture variance in whole fields by detecting the linear combinations so that the components, which were orthogonal to and not correlated with each other, were divided. The repeatability of data was also verified by the PCA.

### Screening of DEGs

GEO2R (16) is a system for online analysis of data in GEO. This tool system runs in R language based on two R packages, GEOquery and limma. The former is used for data reading, and the latter is used for calculation. DEGs between the AF blood samples and the AFST blood samples were screened by GEO2R. *P* ≤ 0.05 was defined as the cut-off criterion. When the DEGs were not annotated with the gene symbols, they were excluded. The DEGs were presented with volcano maps, which were drawn by a volcano plotting tool (https://shengxin.ren) based on R language. Circos (17) was one useful tool to find the overlapping genes based on their shared pathways or functions. Finding the overlapping DEGs between two datasets was performed by Circos. An online Venn tool (http://bioinformatics.psb.ugent.be/webtools/Venn/) was used to apply a VENN diagram to identify the overlap between GSE66724 and GSE58294 and obtain common DEGs.

### Functional Annotation for DEGs via DAVID

Gene Ontology (GO) (18) is a database created by the Gene Ontology Consortium, which consists of a set of predefined GO terms that define and describe the functions of genes and proteins in a variety of species and can be updated as research progresses. The Gene Ontology can be divided into three parts: cellular component (CC), biological process (BP), and molecular function (MF). A protein or gene could find its corresponding GO number through ID matching or sequence annotation, and the GO number could correspond to Term, namely functional category or cell location. KEGG (Release 91.0, July 1, 2019, https://www.kegg.jp/kegg/) (19) is an open database resource for easily understanding utilities and high-level functions of biological systems in the organism, the ecosystem, and the cell from molecular-level information, especially using the large-scale datasets generated from genome sequencing technologies. The Database for Annotation, Visualization, and Integrated Discovery (DAVID) (version: v6.8, https://david.ncifcrf.gov/summary.jsp) (20, 21) provides a well-rounded set of annotation tools for function and pathway enrichment analysis so that the investigators can easily understand the biological meaning of a large list of DEGs. For the given list of DEGs, DAVID tools were able to identify enriched biological themes, particularly GO terms, and visualize genes on BioCarta and KEGG pathway maps.

### Function and Pathway Enrichment Analysis by Metascape

Metascape (http://metascape.org/gp/index.html#/main/step1) (22) is a powerful annotation analysis tool for gene function that can help researchers apply popular bioinformatics analysis methods to the analysis of batch genes and proteins so as to realize the cognition of gene or protein functions. It can annotate a large number of genes or proteins, perform enrichment analysis, and construct protein-protein interaction networks. It integrates several authoritative functional databases such as GO, KEGG, and Uniprot to analyze not only human data, but also data from many other species, and to analyze not only a single data set, but also multiple gene sets simultaneously. The Matascape was used to complete the research's function and pathway enrichment analysis.

### Enrichment Analysis by Gene Set Enrichment Analysis (GSEA)

Gene Set Enrichment Analysis (GSEA) (http://software.broadinstitute.org/gsea/index.jsp) (23, 24) is a powerful computational method that determines whether *a priori* defined set of genes manifests statistically significant differences between two states. Using a predefined set of genes that is usually derived from functional notes or results of previous experiments, GSEA can rank the genes by the degree of differential expression in the two samples, and then check to see if the predefined set of genes is enriched at the top or bottom of the list. Gene set enrichment assays detect changes in the expression of gene sets rather than individual genes and thus can include these subtle changes in expression, with better results expected. GSEA does not need to specify a clear threshold of differentially expressed genes and the algorithm analyzes the overall trend according to the actual situation. GSEA analysis was carried out to perform the GO and KEGG enrichment analysis. GSEA would be conducted on the sequenced genes of AF and AFST blood samples after importing reference function sets, gene annotation files, and all gene data of both AF and AFST blood samples.

### The Construction of Protein–Protein Interaction (PPI) Network, Screening of the Significant Module, and Identification of Hub Genes

An online database, the Search Tool for the Retrieval of Interacting Genes (STRING) (25) (http://string-db.org), can trace and predict the protein–protein interaction (PPI) network after the common DEGs are imported into the database. The STRING was used to construct a PPI network of DEGs. The Cytoscape (version 2.8) (26), an open visualization software tool, was used to visualize this network. The Molecular Complex Detection tool (MCODE) (version 1.5.1) (27), a plug-in of Cytoscape, could screen and identify the most significant module in the PPI network, and the criteria was that MCODE scores >5, the degree of cut-off = 2, node score cut-off = 0.2, k-score = 2, and maximum depth = 100. Furthermore, once the degree was more than 10, the cytoHubba (28), a plug-in of Cytoscape, could identify the hub genes.

### The Correlation Analysis Between the Hub Genes and AFST

Pearson's correlation test was performed to complete the correlation analysis of the hub genes. The mapping of heatmaps, which could present the correlation among the hub genes, was completed by the R language. Spearman's correlation and multiple linear regression analyses between AFST and relevant gene expression were also carried out.

### Enrichment Analysis, Expression Analysis, and Diagnostic Analysis of Hub Genes

The GO and KEGG enrichment analyses for hub genes were completed via the DAVID tool, and the bubble diagrams were drawn by R language. Two heatmaps hub genes' expression level were visualized with R language. Finally, the receiver operator characteristic (ROC) curve analysis was performed to determine the usefulness of these hub genes in predicting AFST. The SPSS software (version 21.0; IBM; New York; America) was used to conduct a statistical analysis. A *P* ≤ 0.05 was considered statistically significant.

### Identification of Hub Genes Associated With Cardiovascular and Nervous Diseases

The comparative toxicogenomics database (http://ctdbase.org/) (29) was used to identify the integrated chemical–disease, chemical–gene, and gene–disease interactions to predict novel associations and generate expanded networks. The relationships between gene products and cardiovascular and nervous diseases were analyzed by the comparative toxicogenomics database.

## Results

### Validation of Data

**Figure 1:**
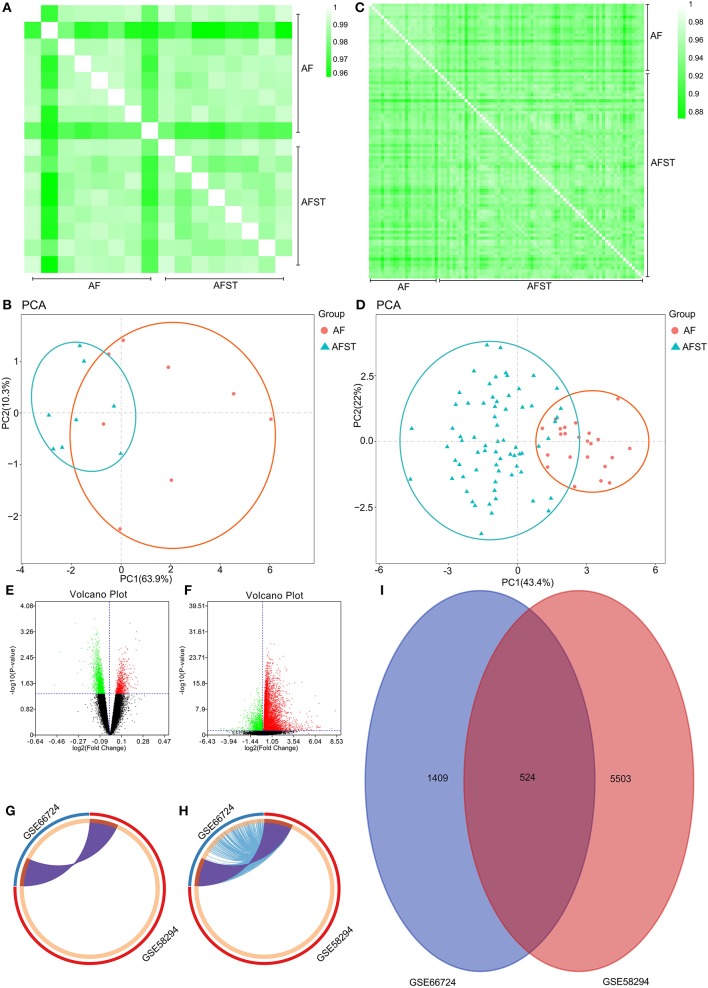
Validation of data, and the identification of DEGs between AF and AFST samples. **(A)** Pearson's correlation test for GSE66724 dataset. **(B)** The principal component analysis for GSE66724. **(C)** Pearson's correlation test for GSE58294 dataset. **(D)** After performing the principal component analysis, the repeatability of data in GSE58294 was fine. **(E)** The DEGs between AF and AFST blood samples in the GSE66724 were presented in the volcano plots. **(F)** The DEGs in the GSE58294 were presented in the volcano plots. **(G)** The overlapping DEGs between GSE66724 and GSE58294 were presented by Circos at gene level. **(H)** The overlapping DEGs between GSE66724 and GSE58294 were presented by Circos at the shared term level. **(I)** The VENN diagram manifested 524 DEGs common to both datasets.

Pearson's correlation test shows that there are powerful correlations among samples in the AF group, and that there are also powerful correlations among samples in AFST group in the GSE66724 dataset (Figure 1A). After performing the principal component analysis, the repeatability of data in GSE66724 was fine (Figure 1B). Pearson's correlation test shows that there are powerful correlations among samples in the AF group, and that there are also powerful correlations among samples in AFST group in the GSE58294 dataset (Figure 1C). After performing the principal component analysis, the repeatability of data in GSE58294 was fine (Figure 1D).

### The Identification of DEGs Between AF and AFST Samples

Through the GEO2R analysis, the DEGs between AF and AFST blood samples in the GSE66724 were presented in the volcano plot (Figure 1E) along with DEGs in the GSE58294 (Figure 1F). The overlapping DEGs between GSE66724 and GSE58294 were presented by Circos not only at gene level (Figure 1G), but also at the shared term level (Figure 1H). The VENN diagram showed that there was a total of 524 DEGs common to both datasets (Figure 1I).

### GO and KEGG Functional Annotation for DEGs via DAVID and Metascape

**Figure 2:**
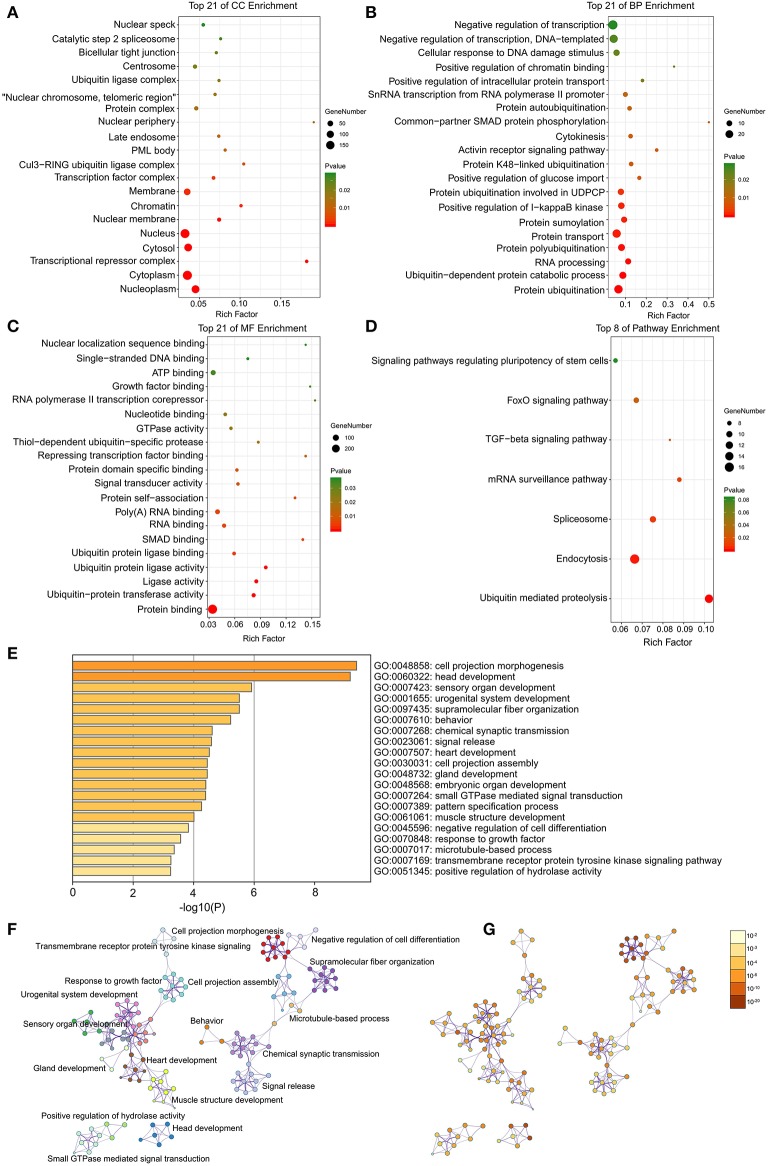
The enrichment analysis of DEGs by DAVID and Metascape. Detailed information relating to changes in the **(A)** CC, **(B)** BP, **(C)** MF, and **(D)** KEGG analysis for hub genes. **(E)** Heatmap of enriched terms across the input differently expressed gene lists, colored by *p*-values, via the Metascape. **(F)** Network of enriched terms colored by cluster identity, where nodes that share the same cluster identity are typically close to each other. **(G)** Network of enriched terms colored by *p*-value, where terms containing more genes tend to have a more significant *p*-value.

Through DAVID analysis, the results of the GO analysis showed that variations in DEGs linked with CC were mainly enriched in nucleoplasm and ubiquitin ligase complex (Figure 2A). Variations in DEGs linked with BP were significantly enriched in protein ubiquitination and ubiquitin-dependent protein catabolic process (Figure 2B). With regard to MF, DEGs were significantly enriched in ubiquitin-protein transferase activity, ubiquitin protein ligase activity, and ubiquitin protein ligase binding (Figure 2C). Analysis of KEGG pathways indicated that the top canonical pathways associated with DEGs was ubiquitin mediated proteolysis (Figure 2D).

Furthermore, the functional enrichment analysis with Metascape found that the DEGs between AFST and AF blood samples were significantly enriched in the head development and heart development (*P* < 0.05, Figures 2E–G).

### Enrichment Analysis Through GSEA

**Figure 3:**
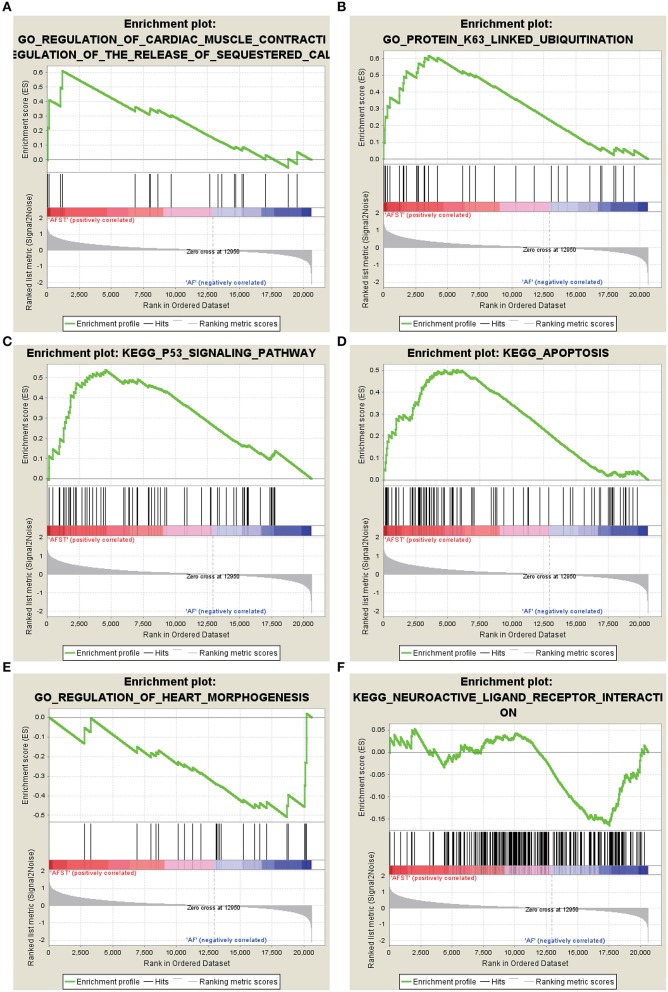
The six enrichments for up-regulated and down-regulated gene sets in the significant order (size of NES). **(A)** Enrichment plot: GO_REGULATION_OF_CARDIAC_MUSCLE_CONTRACTION_BY_REGULATION_OF_THE_RELEASE_OF_SEQUESTERED_CALCIUM_ION. **(B)** Enrichment plot: GO_PROTEIN_K63_LINKED_UBIQUITINATION. **(C)** Enrichment plot: KEGG_P53_SIGNALING_PATHWAY. **(D)** Enrichment plot: KEGG_APOPTOSIS. **(E)** Enrichment plot: GO_REGULATION_OF_HEART_MORPHOGENESIS. **(F)** Enrichment plot: KEGG_NEUROACTIVE_LIGAND_RECEPTOR_INTERACTION.

After implementing the GSEA, the enrichments for upregulated gene sets in the significant order (size of NES) are “GO_REGULATION_OF_CARDIAC_MUSCLE_CONTRACTION_BY_REGULATION_OF_THE_RELEASE_OF_SEQUESTERED_CALCIUM_ION” (Figure 3A), “GO_PROTEIN_K63_LINKED_UBIQUITINATION” (Figure 3B), “KEGG_ P53_SIGNALING_PATHWAY” (Figure 3C), and “KEGG_APOPTOSIS” (Figure 3D), and the enrichments for downregulated gene sets in the significant order (size of NES) are “GO_REGULATION_OF_HEART_MORPHOGENESIS” (Figure 3E) and “KEGG_NEUROACTIVE_LIGAND_RECEPTOR_INTERACTION” (Figure 3F). GSEA also showed that the enrichment gene sets in AFST were mainly related to cardiac muscle, ubiquitination, apoptosis, heart, and neuroactive ligand receptor interaction (Table 2).

**Table 2:** GO and KEGG enrichment analysis of DEGs in AFST using GSEA.

**Gene set name**	**SIZE**	**ES**	**NES**	***P*-value**
**Up-regulated**				
GO_REGULATION_OF_CARDIAC_MUSCLE_CONTRACTION_ BY_REGULATION_OF_THE_RELEASE_OF_SEQUESTERED_CALCIUM_ION	19	0.610	1.898	0.004
GO_PROTEIN_K63_LINKED_UBIQUITINATION	34	0.617	1.796	0.000
KEGG_P53_SIGNALING_PATHWAY	64	0.539	1.646	0.002
KEGG_APOPTOSIS	82	0.503	1.626	0.014
**Down-regulated**				
GO_REGULATION_OF_HEART_MORPHOGENESIS	26	−0.5	−1.6	0.042
KEGG_NEUROACTIVE_LIGAND_RECEPTOR_INTERACTION	252	−0.16	−0.75	0.771

*AFST, atrial fibrillation with stroke; ES, enrichment score; NES, normalized enrichment score*.

### The Construction of PPI and Module Networks, and Selection of Hub Genes

**Figure 4:**
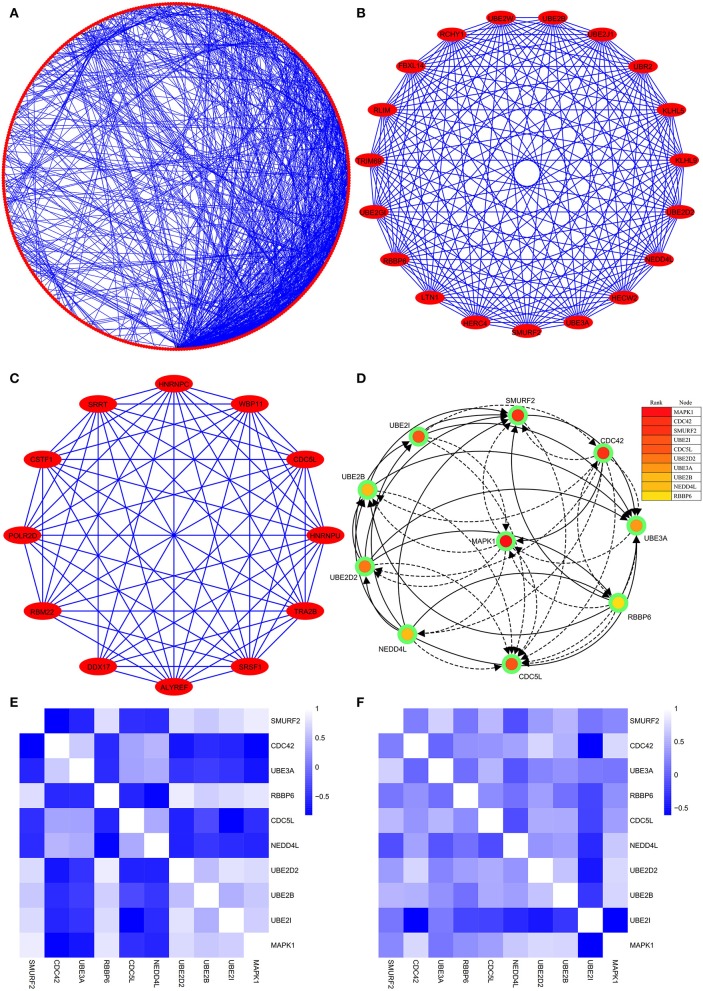
The PPI network of DEGs, two significant modules, hub genes network, and the correlation analysis among hub genes. **(A)** The PPI network consists of 1,162 edges and 413 nodes. **(B)** One module includes a total of 171 edges and 19 nodes. **(C)** Another significant module network consists of 63 edges and 12 nodes. **(D)** The hub genes network (*SMURF2, CDC42, UBE3A, RBBP6, CDC5L, NEDD4L, UBE2D2, UBE2B, UBE2I*, and *MAPK1*). **(E)** The heatmap manifests that there are strong correlations among the all hub genes in the GSE66724. **(F)** The strong correlations among hub genes were also verified in the GSE58294.

The PPI network consists of 1,162 edges and 413 nodes (Figure 4A). There were two significant modules screened from PPI network. One module included a total of 171 edges and 19 nodes (Figure 4B), and another module network consisted of 63 edges and 12 nodes (Figure 4C). Ten hub genes (*SMURF2, CDC42, UBE3A, RBBP6, CDC5L, NEDD4L, UBE2D2, UBE2B, UBE2I*, and *MAPK1*) were identified from the PPI network when the degrees ≥10 (Figure 4D). Summaries for the gene symbols, full names, and function of 10 hub genes are shown in Table 3.

**Table 3:** Summaries for the function of 10 hub genes.

**No**.	**Gene symbol**	**Full name**	**Function**
1	*SMURF2*	SMAD specific E3 ubiquitin protein ligase 2	Among its related pathways are Signaling by Hedgehog and Transcriptional activity of SMAD2/SMAD3-SMAD4 heterotrimer. Gene Ontology (GO) annotations related to this gene include identical protein binding and ubiquitin–protein transferase activity
2	*CDC42*	Cell division cycle 42	Required for DOCK10-mediated spine formation in Purkinje cells and hippocampal neurons. Facilitates filopodia formation upon DOCK11-activation. Also plays a role in phagocytosis through organization of the F-actin cytoskeleton associated with forming phagocytic cups
3	*UBE3A*	Ubiquitin protein ligase E3A	As a regulator of synaptic development by mediating ubiquitination and degradation of ARC
4	*RBBP6*	RB binding protein 6, ubiquitin ligase	May play a role as a scaffold protein to promote the assembly of the p53/TP53-MDM2 complex, resulting in increase of MDM2-mediated ubiquitination and degradation of p53/TP53; may function as negative regulator of p53/TP53, leading to apoptosis
5	*CDC5L*	Cell division cycle 5 like	DNA-binding protein involved in cell cycle control. May act as a transcription activator. The PRP19-CDC5L complex may also play a role in the response to DNA damage (DDR)
6	*NEDD4L*	Neural precursor cell expressed, developmentally down-regulated 4-like, E3 ubiquitin protein ligase	Promotes ubiquitination and degradation of SGK1 and TNK2. Ubiquitinates BRAT1 and this ubiquitination is enhanced in the presence of NDFIP1. Plays a role in dendrite formation by melanocytes. Involved in the regulation of TOR signaling
7	*UBE2D2*	Ubiquitin conjugating enzyme E2 D2	Mediates the selective degradation of short-lived and abnormal proteins. Functions in the E6/E6-AP-induced ubiquitination of p53/TP53
8	*UBE2B*	Ubiquitin conjugating enzyme E2 B	May be involved in neurite outgrowth
9	*UBE2I*	Ubiquitin conjugating enzyme E2 I	Essential for nuclear architecture and chromosome segregation
10	*MAPK1*	Mitogen-activated protein kinase 1	Diseases associated with MAPK1 include chromosome 22Q11.2 deletion syndrome, distal and retrograde amnesia

### Strong Correlation Between the Hub Genes and AFST

Through analyzing the expression data of 10 hub genes in the GSE66724, the strong correlations among the all hub genes were found (Figure 4E). Furthermore, the strong correlations were also verified in the GSE58294 (Figure 4F). Through the Spearman correlation coefficient, MAPK1 (ρ = 0.705, *p* = 0.000) and UBE2D2 (ρ = 0.707, *p* = 0.000) were significantly correlated with AFST (Table 4). In the multivariate linear regression model, holding all other variables at a fixed value, the natural logarithmic AFST remained associated with MAPK1 and UBE2D2 (*p* < 0.05) (Table 4).

**Table 4:** The correlation and linear regression analysis between AFST and relevant gene expression.

**Gene symbol**	**AFST**				
	**Spearman correlation** **coefficient**		**Multiple linear regression**		
	*****ρ***^**a**^**	***p*-value**	*****β***^**b**^**	***p*-value**	**VIF**
SMURF2	0.355	0.001^***^	−0.110	0.347	3.247
CDC42	0.604	0.000^***^	0.054	0.621	3.182
UBE3A	0.360	0.000^***^	0.160	0.043^*^	2.340
RBBP6	0.372	0.000^***^	0.052	0.306	1.332
CDC5L	0.614	0.000^***^	0.196	0.037^*^	2.319
NEDD4L	0.150	0.154	−0.110	0.020^*^	2.851
UBE2D2	0.707	0.000^***^	0.452	0.012^*^	3.352
UBE2B	0.518	0.000^***^	−0.037	0.723	3.484
UBE2I	−0.521	0.000^***^	−0.073	0.086	2.184
MAPK1	0.705	0.000^***^	0.342	0.014^*^	8.204

a Spearman correlation coefficient between AFST and relevant characteristics; ρ, Spearman correlation coefficient.

b Multiple linear regression analysis, β, parameter estimate; AFST, atrial fibrillation with stroke.

VIF, Variance Inflation Factor.

* P ≤ 0.05;

*** *P ≤ 0.001*.

### Functional Enrichment Analysis of Hub Genes

**Figure 5:**
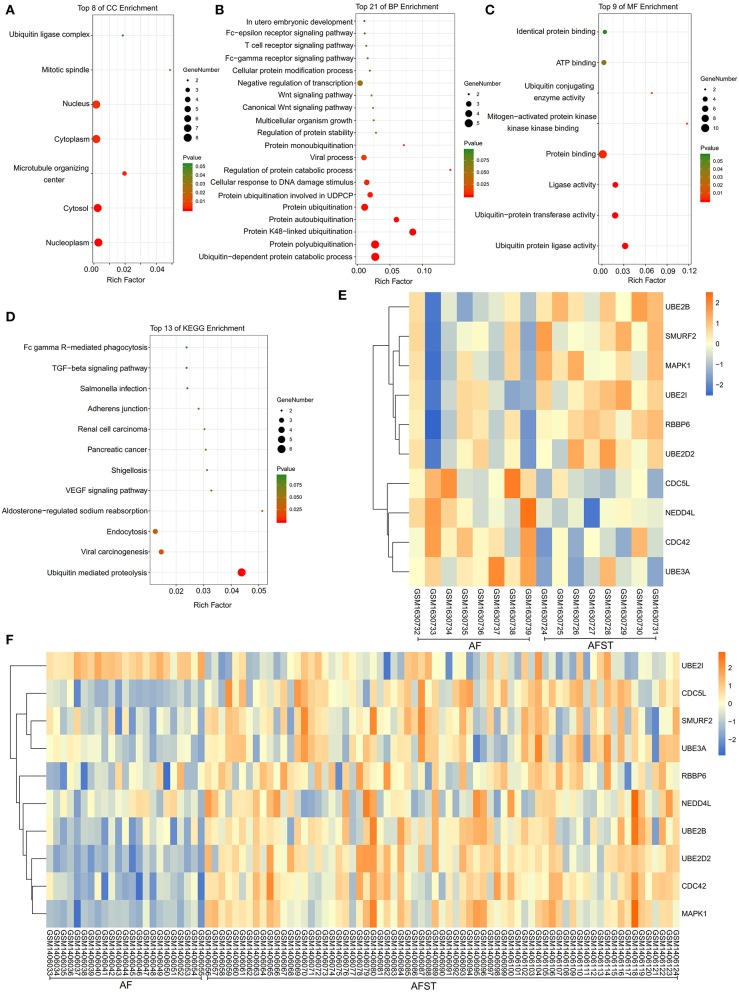
The enrichment analysis and expression level analysis of the hub genes. Detailed information relating to changes in the **(A)** CC, **(B)** BP, **(C)** MF, and **(D)** KEGG analysis for hub genes. **(E)** The comparison of expression level of hub genes between AF and AFST samples in the GSE66724. **(F)** The comparison of expression level of hub genes between AF and AFST samples in the GSE58294.

The results of DAVID showed that variations in CC of hub genes were mainly enriched in ubiquitin ligase complex and nuclei (Figure 5A). Variations in the BP of hub genes were significantly enriched in protein ubiquitination, protein K48-linked ubiquitination, and ubiquitin-dependent protein catabolic process (Figure 5B). Variations in the MF of hub genes were significantly enriched in ubiquitin protein ligase activity, ubiquitin–protein transferase activity, and ubiquitin conjugating enzyme activity (Figure 5C). Analysis of KEGG pathways showed that hub genes were mainly enriched in ubiquitin-mediated proteolysis (Figure 5D).

### The Expression Level of Hub Genes in the AF and AFST Blood Samples

One heatmap showed the expression level of hub genes in the GSE66724. When compared with the AF blood samples, the expression of *MAPK1* and *UBE2D2* were upregulated in the AFST blood samples (Figure 5E). Another heatmap presented the expression level of hub genes in the GSE58294. When compared with the AF blood samples, the expression of *MAPK1* and *UBE2D2* were also upregulated in the AFST blood samples (Figure 5F).

### Hub Genes Could Be Used to Predict AFST Specifically and Sensitively via the ROC Curve Analysis

**Figure 6:**
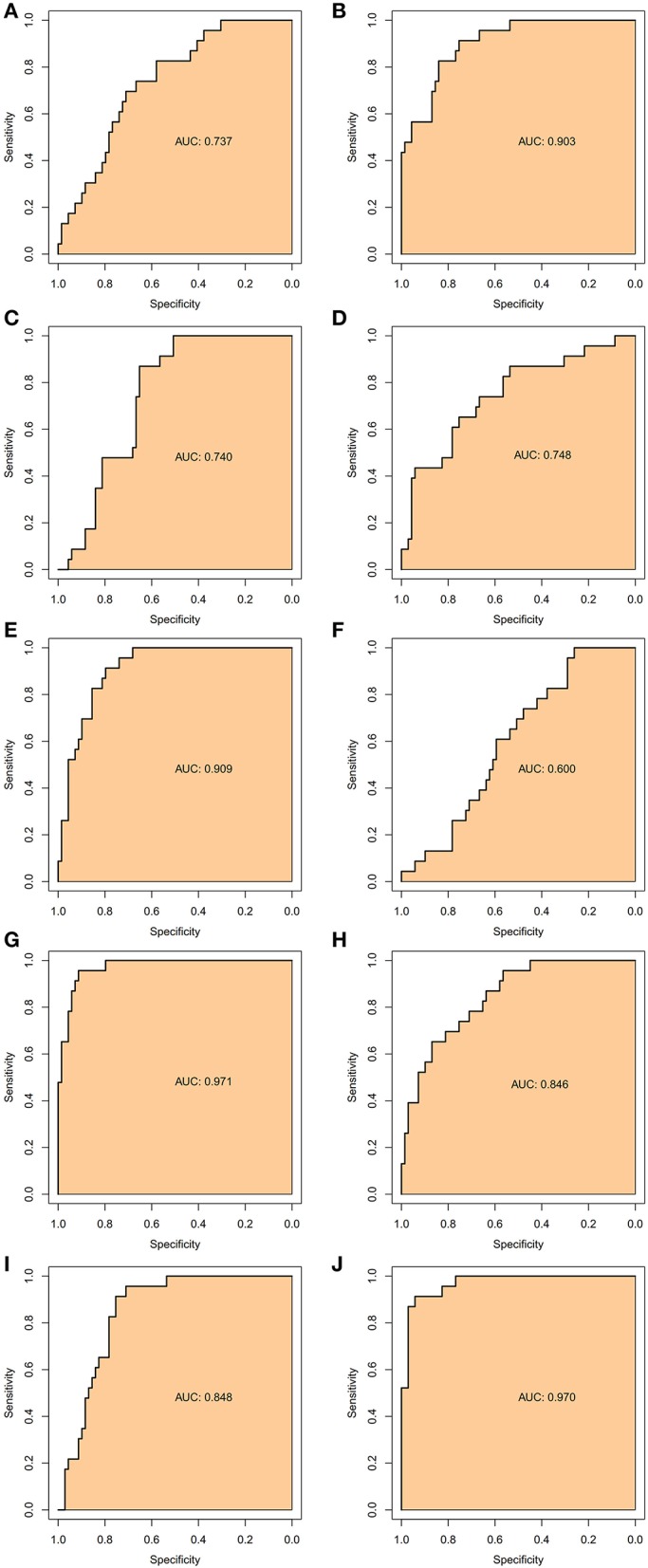
The receiver operator characteristic curves of the hub gene for AFST. **(A)** *SMURF2*, **(B)** *CDC42*, **(C)** *UBE3A*, **(D)** *RBBP6*, **(E)** *CDC5L*, **(F)** *NEDD4L*, **(G)** *UBE2D2*, **(H)** *UBE2B*, **(I)** *UBE2I*, and **(J)** *MAPK1*.

To verify the accurate thresholds of hub genes to predict AFST, the ROC curves was constructed. The expression of *MAPK1* and *UBE2D2* were significantly associated with a diagnosis of AFST (0.7 < AUC <1, *P* ≤ 0.05) (Table 5, Figure 6).

**Table 5:** Receiver operator characteristic curve analysis of hub gene expression for AFST.

**Gene symbol**	**AFST**			
	**AUC**	***P-*value**	**95% CI**	**ODT**
SMURF2	0.737	0.001^***^	0.629–0.844	2.382
CDC42	0.903	0.000^***^	0.839–0.967	2.807
UBE3A	0.740	0.001^***^	0.642–0.838	0.549
RBBP6	0.748	0.000^***^	0.629–0.867	1.225
CDC5L	0.909	0.000^***^	0.851–0.967	1.516
NEDD4L	0.600	0.153	0.481–0.719	0.813
UBE2D2	0.971	0.000^***^	0.942–1.000	3.251
UBE2B	0.846	0.000^***^	0.762–0.929	2.910
UBE2I	0.848	0.000^***^	0.770–0.925	−1.223
MAPK1	0.970	0.000^***^	0.938–1.000	2.228

AUC, area under curve; ^max^ the maximum of AUC;

*significant variables; ODT, optimal diagnostic threshold; AFST, atrial fibrillation with stroke.

*** *P ≤ 0.001*.

### Identification of Hub Genes

**Figure 7:**
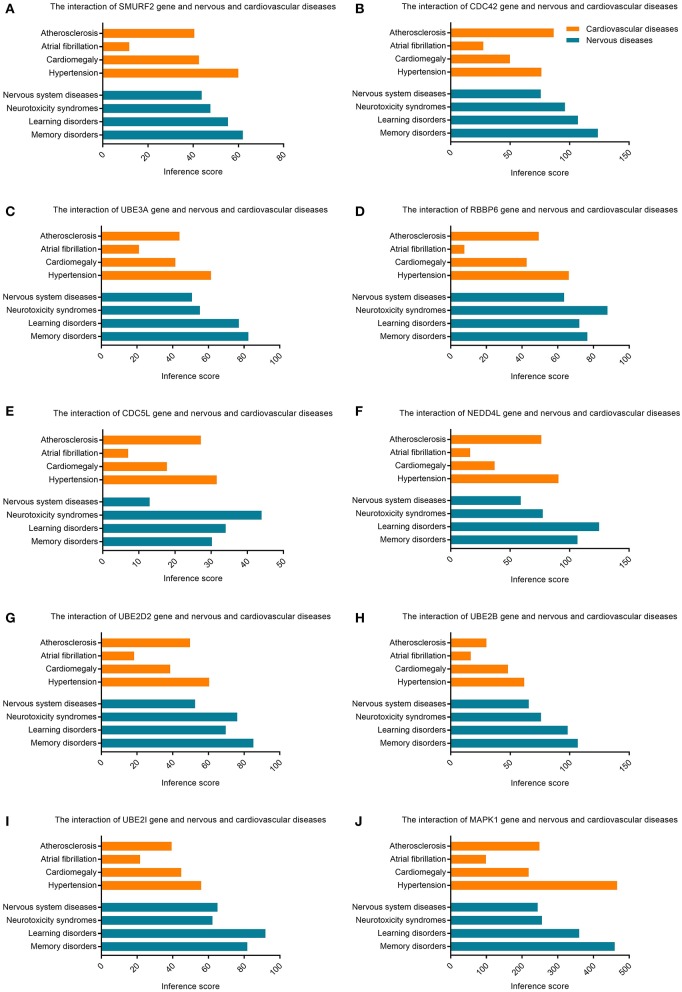
Relationship to cardiovascular or nervous diseases related to hub genes based on the CTD database. **(A)** *SMURF2*, **(B)** *CDC42*, **(C)** *UBE3A*, **(D)** *RBBP6*, **(E)** *CDC5L*, **(F)** *NEDD4L*, **(G)** *UBE2D2*, **(H)** *UBE2B*, **(I)** *UBE2I*, and **(J)** *MAPK1*.

The CTD database showed that hub genes targeted several cardiovascular and nervous diseases. This data is shown in Figure 7.

## Discussion

Atrial fibrillation (AF) increases the risk of ischemic stroke, with an incidence of 1.92% a year. AF patients' risk of ischemic stroke is four to five times that of non-AF patients. Ischemic strokes have a nearly 20% mortality rate and 60% disability rate (30). Therefore, it is of great clinical significance to explore the mechanisms of strokes caused by AF and to search for molecular targets of diagnosis and even treatment (11). Bioinformatics technology has been widely used to find genetic changes during the occurrence and development of diseases, which is a reliable means of finding a target for the diagnosis or treatment of diseases (31).

Our own bioinformatics analysis showed that the ten genes *SMURF2, CDC42, UBE3A, RBBP6, CDC5L, NEDD4L, UBE2D2, UBE2B, UBE2I*, and *MAPK1* were significantly and highly expressed in patients with AF complicated by stroke when compared with patients with simple AF without stroke. Therefore, we speculate that these highly expressed genes, most prominently *MAPK1* and *UBE2D2*, are likely to be involved in incidences of AF complicated by stroke.

*MAPK1* (mitogen-activated protein kinase 1) is mainly involved in affecting the activity of protein serine/threonine kinase, protein phosphorylation, regulating gene expression, and apoptosis, which can participate in the occurrence and development of various diseases (9). Through bioinformatics and microarray technology, Si et al. found that *MAPK1* is involved in the chemotherapy tolerance of breast carcinoma, providing new paths for mechanism research and targeted therapy of chemotherapy tolerance (32). Through bioinformatics analysis, Xu et al. (33) found that *MAPK1* is involved in the drug resistance of ovarian carcinoma. Through experiments, Xu et al. further confirmed the expression of *MAPK1* in ovarian carcinoma drug-resistant cells, providing evidence for the drug resistance mechanism and targeted therapy of tumors (33). Through bioinformatics analysis, Yang et al. (34) found that *MAPK1* overexpression is associated with the incidence of primary colon adenocarcinoma, suggesting that *MAPK1* may be a target for early diagnosis and treatment of primary colon adenocarcinoma. Wang et al. (35) found that the *MAPK1*-related signaling pathway is involved in the occurrence and development of eclampsia, which may serve as a diagnostic target. Mali et al. (36) found that the stromal interacting molecule-1 may participate in the occurrence of myocardial infarction through MAPK, oxidative stress, and apoptosis, providing a new idea for the mechanism research and treatment of myocardial infarction. Through bioinformatics analysis, Zhu et al. (9) found that *MAPK1* can mediate the autophagy of endothelial progenitor cells and participate in the occurrence and development of coronary atherosclerotic heart disease through the mTOR signaling pathway.

At the same time, more and more studies suggest that MAPK is an important regulator of ischemic and hemorrhagic cerebrovascular disease. Through microarray technology, Li et al. (37) found multiple biomarker molecules related to ischemic stroke and found that the MAPK signaling pathway may be involved in the occurrence and development of ischemic stroke. Huang et al. (38) found that the blood let-7e-5p may be a diagnostic target for ischemic stroke through bioinformatics analysis. Further analysis showed that MAPK is involved in the occurrence and development of ischemic stroke, suggesting that MAPK may serve as a therapeutic target. Eyileten et al. (39) summarized the possible diagnostic biomarkers of ischemic stroke and thought that *MAPK1* could be a potential diagnostic and therapeutic target. Hayashi et al. (40) found that the *MAPK1* signaling pathway is involved in autophagy, which is associated with myocardial infarction and AF (41, 42). We found that *MAPK1* is highly expressed in patients with AF complicated by cerebral infarction compared with patients with AF alone. Therefore, we speculate that *MAPK1* is involved in the onset of AF complicated by cerebral infarction through multiple mechanisms. Due to the existence of eddy currents in the atria of patients with AF, *MAPK1* can promote the detachment and metastasis of atrial thrombus by regulating apoptosis, autophagy, inflammatory stress, and other elements, ultimately causing a stroke. In future studies, it is worth considering how *MAPK1* may be a target for the early diagnosis and treatment of AF complicated by stroke.

*UBE2D2* (ubiquitin conjugating enzyme E2 D2) is mainly involved in affecting the binding of ubiquitin protein ligases and the activity of ubiquitin protein transferase (43). *UBE2D2* plays an important role in tissues and cells, where it can participate in the dissolution of tissues through the ubiquitination enzyme system (44). Geisler et al. (45) found that *UBE2D2*-mediated autophagy is involved in the progression of Parkinson's disease. Lee et al. (46) found that the expression of *UBE2D2* is associated with the prognosis of patients with colorectal cancer (CRC), suggesting that *UBE2D2* could be used as one of the prognostic indicators of CRC. Peng et al. (47, 48) argued that autophagy involved with *UBE2D2* has a significant impact on the internal environment and can promote the formation and degradation of ubiquitinated aggregates through autophagy. More recent research on *UBE2D2* provides new ideas for the mechanism research and targeted therapy of autophagy-related diseases.

There is also a correlation between ubiquitin protease activity, autophagy, and the onset of AF and ischemic stroke (49). Hou et al. (42) contended that moderate autophagy can remove damaged organelles, thereby protecting cells from various damages. However, excessive autophagy can cause the degradation of normal cell and tissue contents, which results in tissue and organ damage. In animal models of ischemic brain injury, autophagy can activate and participate in the death of neurons, suggesting that the regulating of autophagy may be a new treatment approach for ischemic stroke (41, 42). We found that *UBE2D2* is highly expressed in patients with AF complicated by cerebral infarction, compared with patients with AF alone. We speculate that in incidences of AF complicated by cerebral infarction, *UBE2D2* may be involved by affecting the activity of ubiquitin protease and autophagy. *UBE2D2* can also induce the detachment of intra-atrial thrombus by regulating the activity of ubiquitin enzyme and autophagy, thereby increasing the incidence of stroke (45). We thus suggest that *UBE2D2* can be a potential diagnostic and therapeutic target for patients with AF complicated by stroke. The relevant mechanism is worth further exploration.

In addition, gene expression could be different according to the stroke phase, and Oh et al. (50) researched the blood genomic profiling of the peripheral blood in the acute phase of ischemic stroke. Stamova et al. (12) studied the gene expression of the hyperacute stroke. We found that there were differences in genomic profiling between hyperacute and acute stoke. Furthermore, a stroke might cause a range of biochemical reactions in the body, from the level of genes to the level of proteins (51). Therefore, the variations in gene expression might be the consequence rather than the cause of a stroke.

Despite this study's rigorous bioinformatics analysis, there were still some outlooks in this study. First, the paper focused on the genomic profiling of a stroke caused by AF. However, it would also be useful to investigate gene expression in patients with strokes due to other causes than AF and it will be defined as the next step to explore the genes of stroke without AF. Second, this study lacks further mechanism validation. The reliability of the conclusion can be improved through animal experiments and clinical sample comprehensive verification. Third, there is increasing evidence that strokes may occur in the context of atrial cardiopathy, even in the absence of clinically overt AF (52, 53). It is, however, not completely understood whether atrial cardiopathy is only a marker and a surrogate of AF or if it can be an independent stroke risk factor. Further, we do not know exactly whether the pathophysiological mechanisms underlying AF and atrial cardiopathy are similar and how much they overlap. Accordingly, the analysis of gene expression should be extended to patients meeting the criteria of atrial cardiopathy, with and without stroke.

## Conclusion

Bioinformatics analysis can effectively identify the differential genes in patients with AF complicated by cerebral infarction vs. patients with AF alone, especially the *MAPK1* and *UBE2D2* genes. These genes are involved in the incidence of AF complicated by cerebral infarction by affecting multiple signaling pathways, which may serve as the target of early diagnosis or treatment. Our study provides new evidence and ideas for further exploration of the mechanism and treatment of AF complicated by stroke.

## Data Availability Statement

The datasets used and/or analyzed during the current study are available from the corresponding author upon reasonable request.

## Ethics Statement

The data of this research was downloaded from the GEO database, a public website. And all institutional and national guidelines for the care and use of participants were followed.

## Author Contributions

YZ and LM analyzed the data and were major contributors in writing. MH was involved in critically revising the manuscript for important intellectual content. TZ and JY made substantial contributions to research conception and designed the draft of the research process. All authors read and approved the final manuscript.

### Conflict of Interest

The authors declare that the research was conducted in the absence of any commercial or financial relationships that could be construed as a potential conflict of interest.
